# *Akkermansia muciniphila*-Nlrp3 is involved in the neuroprotection of phosphoglycerate mutase 5 deficiency in traumatic brain injury mice

**DOI:** 10.3389/fimmu.2023.1172710

**Published:** 2023-05-23

**Authors:** Yuhua Chen, Junhui Chen, Hong Wei, Kai Gong, Jiao Meng, Tianlin Long, Jianfeng Guo, Jun Hong, Lingjian Yang, Junling Qiu, Kun Xiong, Zhanxiang Wang, Quanhua Xu

**Affiliations:** ^1^ Department of Anatomy and Neurobiology, School of Basic Medical Science, Central South University, Changsha, Hunan, China; ^2^ Department of Neurosurgery, Bijie Traditional Chinese Medical Hospital, Bijie, Guizhou, China; ^3^ Department of Central Laboratory, Xi’an Peihua University, Xi’an, Shaanxi, China; ^4^ Xiamen Key Laboratory of Brain Center, Department of Neurosurgery, Trauma Center, The First Affiliated Hospital of Xiamen University, Xiamen, Fujian, China; ^5^ Department of Rehabilitation Teaching and Research, Xi’an Siyuan University, Xi’an, China; ^6^ School of Chemistry & Chemical Engineering, Ankang University, Ankang, China; ^7^ Department of Cardiology, First Hospital of Northwestern University, Shannxi, China; ^8^ Key Laboratory of Emergency and Trauma, Ministry of Education, College of Emergency and Trauma, Hainan Medical University, Haikou, Hainan, China; ^9^ Hunan Key Laboratory of Ophthalmology, Changsha, Hunan, China

**Keywords:** TBI, gut-microbiota-brain axis, *Akkermansia muciniphila*, Nlrp3, *Pgam5*, neuroprotection

## Abstract

**Introduction:**

Gut-microbiota-brain axis is a potential treatment to decrease the risk of chronic traumatic encephalopathy following traumatic brain injury (TBI). Phosphoglycerate mutase 5 (PGAM5), a mitochondrial serine/threonine protein phosphatase, resides in mitochondrial membrane and regulates mitochondrial homeostasis and metabolism. Mitochondria mediates intestinal barrier and gut microbiome.

**Objectives:**

This study investigated the association between PGAM5 and gut microbiota in mice with TBI.

**Methods:**

The controlled cortical impact injury was established in mice with genetically-ablated *Pgam5* (*Pgam5^−/−^*) or wild type, and WT male mice were treated with fecal microbiota transplantation (FMT) from male *Pgam5^−/−^* mice or *Akkermansia muciniphila* (*A. muciniphila*). Then the gut microbiota abundance, blood metabolites, neurological function, and nerve injury were detected.

**Results:**

Treated with antibiotics for suppressing gut microbiota in *Pgam5^−/−^* mice partially relieved the role of *Pgam5* deficiency in the improvement of initial inflammatory factors and motor dysfunction post-TBI. *Pgam5* knockout exhibited an increased abundance of *A. muciniphila* in mice. FMT from male *Pgam5^−/−^* mice enabled better maintenance of amino acid metabolism and peripherial environment than that in TBI-vehicle mice, which suppressed neuroinflammation and improved neurological deficits, and *A. muciniphila* was negatively associated with intestinal mucosal injury and neuroinflammation post-TBI. Moreover, *A. muciniphila* treatment ameliorated neuroinflammation and nerve injury by regulating Nlrp3 inflammasome activation in cerebral cortex with TBI.

**Conclusion:**

Thus, the present study provides evidence that Pgam5 is involved in gut microbiota-mediated neuroinflammation and nerve injury, with *A. muciniphila*-Nlrp3 contributing to peripheral effects.

## Introduction

With the rapid globalization of the economy and transportation, traumatic brain injury (TBI) has become one of the main causes of disability and death and a huge public problem that threatens human health and life of approximately 70 million people annually. Exemplary traffic laws and health education and advanced neurocritical and medical care can reduce the incidence and mortality of TBI (1–3). Despite the growing awareness about the brain in recent decades, there is still no specific treatment strategy for TBI in clinical practice (4). Because the primary injury of TBI occurs instantaneously, effective intervention is usually not possible. Strengthening the research on the pathogenesis and intervention measures of the secondary injury is potential therapeutic options to improve the outcome of TBI (3, 4). Therefore, it’s important to identify the pathological events that contribute to the progression of secondary injury and peripheral influences that can exacerbate local pathophysiological responses and/or exert neuroprotective effects (5).

Gut-microbiota-brain axis (GMBA) is a potential treatment to decrease the risk of nervous system impairment following TBI (5–7). The GMBA is a complex circuit that operates through the crosstalk of the gut microbiome, enteric nervous system, autonomic nervous system, central nervous system (CNS), neuronal immunology and endocrine signals, and hypothalamic-pituitary axis (7, 8). Generally, in response to TBI, epithelial dysfunction, alterations in the gut microbiota alteration, and aberrant immune responses are commonly observed (7). Some scholars have proposed that TBI led to gastrointestinal tract damage, which in turn drives immune reactions and systemic inflammation which further exacerbates neuroinflammation (9, 10).. Houlden et al. report bacterial population shifts post-TBI, including Bacteroidetes, Firmicutes, and α-Proteobacteria, which are correlated with neurological deficits (11). The perturbations of gut microbiota composition initially occur 24~72 h after TBI, which corresponds to pathophysiology of secondary injury in TBI (11–14). Thus, the gut microbiota and its metabolite composition are potential diagnostic and therapeutic biomarkers for TBI (6, 7). *Akkermansia muciniphila (A. muciniphila)* is the sole Gram-negative emblematic Verrucomicrobia and extensively colonized in the human intestinal mucosa (15, 16) and has been considered a promising candidate for probiotics (17, 18), but its function in TBI has been rarely reported.

Phosphoglycerate mutase 5 (PGAM5), a mitochondrial serine/threonine protein phosphatase, resides in mitochondrial membrane and participates in multiple processes via DRP1, FUNDC1, and BCL-xL phosphorylation, which regulates mitochondrial homeostasis, mitophagy, cell death, metabolism, aging, and inflammation (19–21). Some studies have shown that PGAM5 inhibition improves fat metabolization and prevents severe metabolic stress (22, 23). Zhu et al. report that PGAM5-mediated dephosphorylation of malic enzyme 1 S336 affects lipid metabolism and colorectal tumorigenesis susceptibility (24). Previously, we have verified Pgam5 as a mediator in progression of nerve damage by Drp1 phosphorylation-mediated mitochondrial dysfunction, and *Pgam5* knockout (KO) alleviated inflammatory responses and neurological disorder in mice with TBI (25). Mitochondria in intestinal epithelial cells participates in cellular functions, which mediates intestinal barrier and gut microbiome. Recently, Duan et al. demonstrate drp1 affects gut microbiota and intestinal barrier following hemorrhagic shock by regulating mitochondrial function (26). However, whether PGAM5-mediated gut microbiota is involved in neuronal damage and neuroinflammation has not been determined.

Considering the role of mitochondrial homeostasis in gut microbiota and to further interpret the function of PGAM5 in nerve damage following TBI, the present study investigated the association between Pgam5 and the gut microbiota in mice with TBI. This study provided new insights and revealed pathological changes of acute TBI in *Pgam5* deficient mice and the potential benefits of *A. muciniphila* in mice with TBI, which may help to more accurately identify the potential treatment outcome of TBI.

## Materials and methods

### Animals

All *in vivo* experiments were approved by the Animal Care and Use Committee of Xi’an Peihua University, China, and performed according to the recommendations of the Guide for the Care and Use of Laboratory Animals. C57BL/6N *Pgam5*-deficient and wild type (WT) mice (8 weeks, 20 ± 2 g) were fed with a 12-h/12-h light-dark cycle, along with free activity and foraging (including food and water) and the food is the Lab Mice Diet (SFS9112; Xietong Pharmaceutical Bioengineering Co., LTD, Nanjing, China).

### TBI model

The *in vivo* TBI model was established using a PinPoint PCI3000 device. After anesthesia with sodium pentobarbital (50 mg/kg) and analgesia with 1 mg/kg sustained-release buprenorphine (subcutaneous injection, every three days) and 5 mg/kg carprofen (subcutaneous injection, daily), mice were placed on a brain stereotaxis instrument, then a 5 mm midline incision was made over the skull, and a craniotomy was made on the central aspect of the right parietal bone (25, 27). The device was adjusted to a parameter with a 5.0 m/s velocity, 2 mm depth, and 100 ms dwell time (25, 27). After the operation, skulls were sealed, incisions were closed, and male mice were put in a heated cage with 38°C until they regained consciousness. The sham group was with normal surgical procedures, except for impact. The *in vivo* experiments and tissue samples collection were performed by double-blind experiment. Instead of Study 1~4 being carried out simultaneously, they are carried out one by one. Therefore, only the same batch of animals was used in the data analysis.

### *Akkermansia muciniphila* treatment

*A. muciniphila* was cultured in brain heart infusion broth (Quelab, Canada) supplemented with 0.5% porcine mucin (Sigma-Aldrich) with mild shaking (150 rpm) under the abovementioned conditions for 48 h and until reaching an optical density 600 of 1; then the medium was removed, centrifuged at 3000 rpm for 10 min, and washed twice with anaerobic phosphate-buffered saline (PBS). The mice were gavaged daily for 3 consecutive days with 1 × 10^9^ colony-forming unit (CFU) of *A. muciniphila* in 200 µL sterile PBS or only 200 µL sterile PBS (28).

### Study 1

All WT and *Pgam5^-/-^* sham or TBI male mice were conducted behavioral tests at 1, 3, 7, and 14 days post-TBI (dpt). At 3, 7, and 14 dpt, animals were euthanized, and brain tissues were immediately obtained, frozen in liquid nitrogen or fixed with 4% paraformaldehyde, and stored at –80°C for further analysis.

### Study 2

Before TBI, WT and *Pgam5^-/-^* male mice drank water with ampicillin (1 g/L), vancomycin (500 mg/L), ciprofloxacin (200 mg/L), imipenem plus cilastatin (250 mg/L), and metronidazole (1 g/L) for 2 weeks, after which antibiotic treatment was discontinued and replaced with sterile tap water 72 h before the injury. All WT and *Pgam5^-/-^* sham or TBI mice were conducted behavioral tests. At 3, 7, and 14 dpt, animals were euthanized and brain tissues were obtained for further analysis.

### Study 3

WT mice were randomly divided into sham + vehicle, TBI + vehicle, sham + fecal microbiota transplantation (FMT), and TBI + FMT. About 200 mg of stool was daily collected from male *Pgam5^-/-^* mice and resuspended in bioclean PBS (with 10 ml), and passed through a 20 mm filter to remove large impurities. The supernatant was discarded with 3,000 × g centrifugation, approximately 4 × 10^9^ viable bacteria dissolved in 4 ml sterile PBS and then for gavage. Before TBI, male animals drank water with ampicillin (1 g/L), vancomycin (500 mg/L), ciprofloxacin (200 mg/L), imipenem plus cilastatin (250 mg/L), and metronidazole (1 g/L) for 2 weeks, and then the antibiotic treatment was discontinued and replaced by sterile tap water 72 h before the injury. Mice in the vehicle and FMT groups were given 0.2 mL sterile PBS or FMT with a continuous three-day after model preparation, and the first gavage was 4 h after injury. All WT and *Pgam5^-/-^* sham or TBI mice were conducted behavioral tests. At 3, 7, and 14 dpt, animals were euthanized and brain tissues were obtained for further analysis (at 3 dpt, mice were euthanized 4 h after gavage).

### Study 4

WT male mice were randomly divided into 2 groups: TBI + vehicle and TBI + *A. muciniphila*. Before TBI, animals drank water with ampicillin (1 g/L), vancomycin (500 mg/L), ciprofloxacin (200 mg/L), imipenem plus cilastatin (250 mg/L), and metronidazole (1 g/L) for 2 weeks, and then the antibiotic treatment was discontinued and replaced by sterile tap water 72 h before the injury. Mice in the vehicle and *A. muciniphila* groups were given 0.2 ml sterile PBS or *A. muciniphila* (1 × 10^9^ CFU, with reference to Pang et al. (28)) with a continuous three-day after model preparation, and the first gavage was 4 h after injury. All WT and *Pgam5^-/-^* sham or TBI mice were conducted behavioral tests. At 3, 7, and 14 dpt, animals were euthanized and brain tissues were obtained for further analysis (at 3 dpt, mice were euthanized 4 h after gavage).

### Behavioral tests

The modified neurological severity score (mNSS) and beam walk test were conducted to discuss neurological deficits using a double-blind experiment (25, 27). Behavioral tests were conducted before and 1, 3, 7, and 14 dpt. Before the experiments, all animals underwent behavioral experiments and were found to be normal. The mNSS test consists of multiple items to assess motor, balance, sensory, and reflex functions of mice, the scores for individual measurements that comprise the mNSS. Neurological function is graded from 0 to 18, higher scores implied graver neurological injury, according to the previous study (25, 27). The beam walk test analyzed motor coordination differences and consisted of an overhead wooden beam (5 mm wide, 120 mm length, and 300 mm above table). Animals were put at the end of the beam and it recorded the frequency of foot defects in the right hind limb over 50 steps. Beam walking training was performed 3 d before TBI.

### Enzyme-linked immunosorbent assay analysis

Serum and tissue Tnf-α and Il-1β levels were analyzed using an ELISA kit (PT512 and PI301; Beyotime, Shanghai, China), and D-lactic acid (D-LA) and diamine oxidase (DAO) levels in the serum were also measured (A088-2-1 and H263-1-2; Nanjing Jiancheng Bioengineering Institute, Nanjing, China).

### Histological evaluation

After fixation with 4% paraformaldehyde, tissue paraffin 4 μm slices were conventional dyed with hematoxylin and eosin (H&E) and Nissl staining and immunohistochemistry were conducted to analysis the histopathological condition around the injury site. Nissl staining was conducted by Nissl Staining Solution (Cresyl Violet) (Solarbio Science & Technology Co., Ltd., Beijing, China). The brain sections were incubated with Nlrp3 antibody (ab270449, Abcam, 1:100) overnight at 4°C, incubated with HRP-linked secondary antibody (ASR1651, Abcepta), and stained by DAB solution. Microscopic observation of the histological slides was performed using a light microscope (10× objective). Both Nissl-positive cells in randomly three fields of primary somatosensory cortex around the injury site (parietal cortex) per brain were counted by the ImageJ software (1.4, NIH).

### Western blot analysis

After lysed, the protein concentration was quantified using a bicinchoninic acid protein assay kit (Thermo Scientific). 30 μg protein per lane were loaded on SDS-PAGE gels. After electrophoresis, the proteins were transferred to PVDF membranes. The membranes were blocked with 5% BSA and incubated at 4°C overnight with the appropriate primary antibodies: IbA1(17198S, CST, 1:1,000), Nlrp3 (ab270449, Abcam, 1:1,000), caspase1 (ab179515, Abcam, 1:1,000), caspase1 (p20) (AG-20B-0042-C100, AdipoGen, 1:1,000), Il-1β (31202S, CST, 1:1,000), Gapdh (YM3029, Immunoway,1:5,000), and incubated with horseradish peroxidase-conjugated secondary antibodies (ASR1937 and ASR1651, Abcepta, 1:20,000). Protein bands were visualized by enhanced chemiluminescence (Bio-Rad, Hercules, CA, USA) and images were captured using a ChemiDoc™ MP imaging system (Bio-Rad). All samples were run in parallel with four replicates.

### qRT-PCR analysis

The colon or cortical tissue was homogenized by the homogenizer (Tiangen Bio, Beijing, China) and the total RNA was isolated using Trizol reagent (Invitrogen, Waltham, MA, USA). A HiFi-MMLV cDNA First Strand Synthesis Kit (CW Bio, Beijing, China) was used for reverse transcription. FastFire qPCR PreMix (Tiangen Bio) was used for qRT-PCR analysis on the CFX96TM real-time system (Bio-Rad). The expression of the genes of interest was normalized to the levels of *GAPDH*. Primer sequences were listed as follows: *Nlrp3*: *Nlrp3*: F: 5’-GCTCCAACCATTCTCTGACC-3’, R: 5’-AAGTAAGGCCGGAATTCACC-3’; *Gapdh*: F: 5´-AACTTTGGCATTGTGGAAGG-3´, R: 5´-GGATGCAGGGATGATGTTCT- 3´. Each sample was analyzed and quantified using the 2−ΔΔCq method.

### 16S rRNA gene sequencing analysis

The relevant content is provided by LC-Bio Technology Co., Ltd. (Hangzhou, China). 16S ribosomal RNA (rRNA)-based amplification the V3 and V4 hypervariable regions was conducted, with primers 338F: 5’-ACTCCTACGGGAGGCAGCAG-3’; 806R: 5’-GGACTACHVGGGTWTCTAAT-3’. The Pusion Hot Start Flex 2X Master Mix (M0536; Shanghai Yitao Biological Instrument Co., LTD., Shanghai, China) was used for PCR amplifications prior to 16S rRNA gene sequencing analysis. AMPure XT beads (Beckman Coulter Genomics, Danvers, MA, USA) was used to purify PCR products and Qubit (Invitrogen) was used to quantify PCR products. The Library Quantification Kit for Illumina (Kapa Biosciences, Woburn, MA, USA) was used to assess the size and quantity of the amplicon library. Samples were sequenced on an Illumina MiSeq PE300 platform (Illumina, Inc., CA, USA) (2 × 300 bp), and preliminary analysis were according to the standard protocols of LC-Bio Technology Co., Ltd.

### Metabolite analysis in the serum

Before analysis, the serum samples were thawed on ice and processed to remove the proteins. First, 50 µL serum was removed; Second, 200 µL methanol (precooled at -20° C) was added, vortexed for 60 s, centrifuged at 4° C for 10 min at 12,000 rpm, and transferred supernatants into another 1.5 ml tube; Third, the samples were concentrated and dried under vacuum; Fourth, samples were dissolved with 75 μL 2-chlorobenzalanine (4 ppm)-80% methanol solution and supernatant were filtered (by 0.22-µm membrane) to obtain LC-MS samples. Finally, the rest of the samples were used for ultraperformance liquid chromatography-tandem mass spectrometry detection and preliminary analysis were according to the standard protocols of LC-Bio Technology Co., Ltd.

### Statistical analyses

All the data were prepared as the mean ± standard error of the mean (SEM) and analyzed using the Statistical Package for the Social Sciences statistical software and GraphPad Prism 5 software. For multiple comparisons more than two groups and variate, data were analyzed using two-way ANOVA followed by Bonferroni’s *post hoc* test with normally distributed or by the Kruskal-Walli’s test with non-normally distributed. All data examining groups over time were analyzed by a Repeated Measures ANOVA, and then Tukey’s *post hoc* test was conducted to compare the differences between groups on the same time. Result was considered significant at *p* < 0.05.

## Results

### Elimination of the gut microbiota alleviates the neuroprotective effect of Pgam5 deficiency in mice with TBI

**Figure 1:**
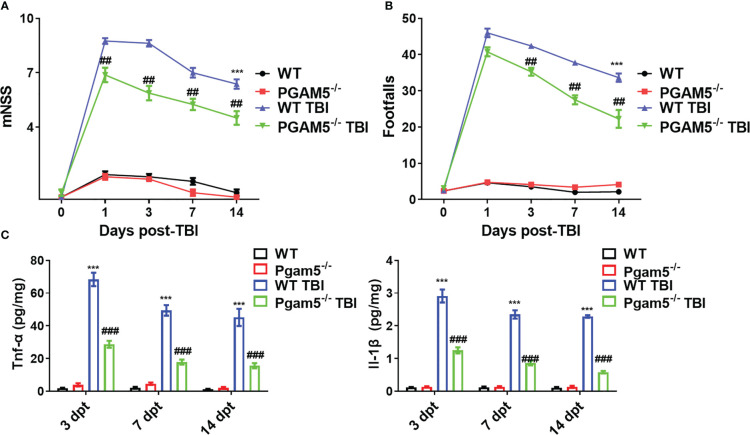
*Pgam5* deficiency shows the neuroprotective effect on TBI mice. After established TBI model in WT and *Pgam5^−/−^* male mice, the behavioral tests and inflammatory cytokine detection were conducted. Behavioral test was analyzed by mNSS **(A)** and beam walk test **(B)** at 1, 3, 7, and 14 dpt, n = 8. **(C)** ELISA analysis of Tnf-α and Il-1β in cortical tissue at 3, 7, and 14 dpt, n = 6. *** *p* < 0.001 *vs.* WT group. ## *p* < 0.01, ### *p* < 0.001, *vs.* WT TBI group. Data shown are means ± SEM.

**Figure 2:**
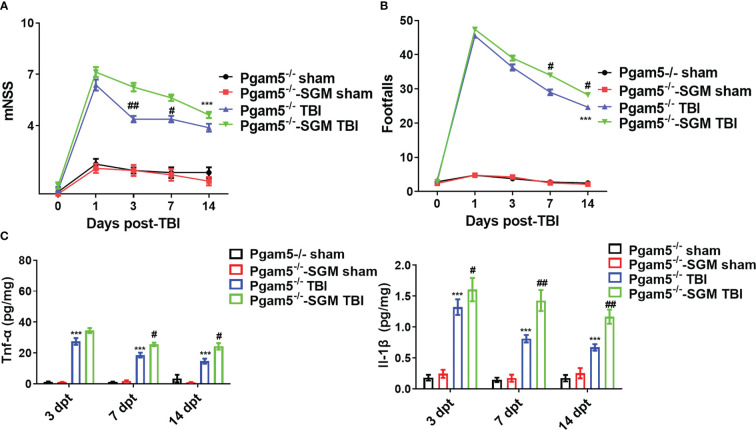
Elimination of gut microbiota alleviates the neuroprotective effect of *Pgam5* deficiency on TBI mice. After treated with antibiotics for suppressing gut microbiota (SGM) for 2 weeks, *Pgam5* ^-/-^ male mice were underwent TBI, and behavioral tests and inflammatory cytokine detection were conducted. Behavioral test was analyzed by mNSS **(A)** and beam walk test **(B)** at 1, 3, 7, and 14 dpt, n = 8. **(C)** ELISA analysis of Tnf-α and Il-1β in cortical tissue at 3, 7, and 14 dpt, n = 6. *** *p* < 0.01 *vs. Pgam5* ^-/-^ sham. # *p* < 0.05, ## *p* < 0.01, *vs. Pgam5^-/-^* TBI group. Data were shown as means ± SEM.

Before the experiments, behavioral tests were conducted, and there were no differences in the mNSS and beam walk between all animals. As shown in **Figure 1**, TBI increased the mNSS and footfalls at 1, 3, 7, and 14 dpt, whereas *Pgam5^-/-^* mice with TBI showed lower mNSS at 1, 3, 7, and 14 dpt and fewer footfalls at 3, 7, and 14 dpt compared with those of WT mice with TBI (*p* < 0.05, **Figures 1A, B**). At 3, 7, and 14 dpt, Tnf-α level and Il-1β level in the cortical tissue were detected. Tnf-α level and Il-1β level peaked at 3 dpt and then gradually decreased in the cortical tissue after TBI (**Figure 1C**), but *Pgam5* KO reduced Tnf-α level and Il-1β level in mice with TBI during this phase (*p* < 0.001, **Figure 1C**). The data suggest *Pgam5* KO is neuroprotective in TBI mice. To eliminate the influence of gut microbiota differences on experimental results, animals were treated with antibiotics for suppressing gut microbiota (SGM) for 2 weeks. Subsequently, *Pgam5^-/-^* mice experienced TBI, and behavioral tests and ELISA analyses were conducted. TBI caused nerve dysfunction following increased mNSS in mice (**Figure 2A**), and motor function impairment in TBI mice was associated with persistent foot faults at 1, 3, 7, and 14 dpt (**Figure 2B**). *Pgam5* KO improved neurological dysfunction after TBI, but SGM treatment mitigated the neuroprotective effect at 7 dpt in *Pgam5^-/–^* SGM mice with TBI (**Figures 2A, B**). Similar phenomena of Tnf-α level and Il-1β level in the cortical tissue were observed that SGM treatment aggravated Tnf-α level and Il-1β level at 7 and 14 dpt, but not Tnf-α at 3 dpt, in *Pgam5^-/–^* SGM TBI mice *vs.* the *Pgam5^-/-^* TBI group (*p* < 0.05, **Figure 2C**). The data showed that the elimination of gut microbiota in *Pgam5^-/-^* male mice partially relieved the role of *Pgam5* deficiency in the improvement of initial inflammatory factors and motor dysfunction post-TBI.

### Pgam5 deficiency alters the gut microbiota in mice

**Figure 3:**
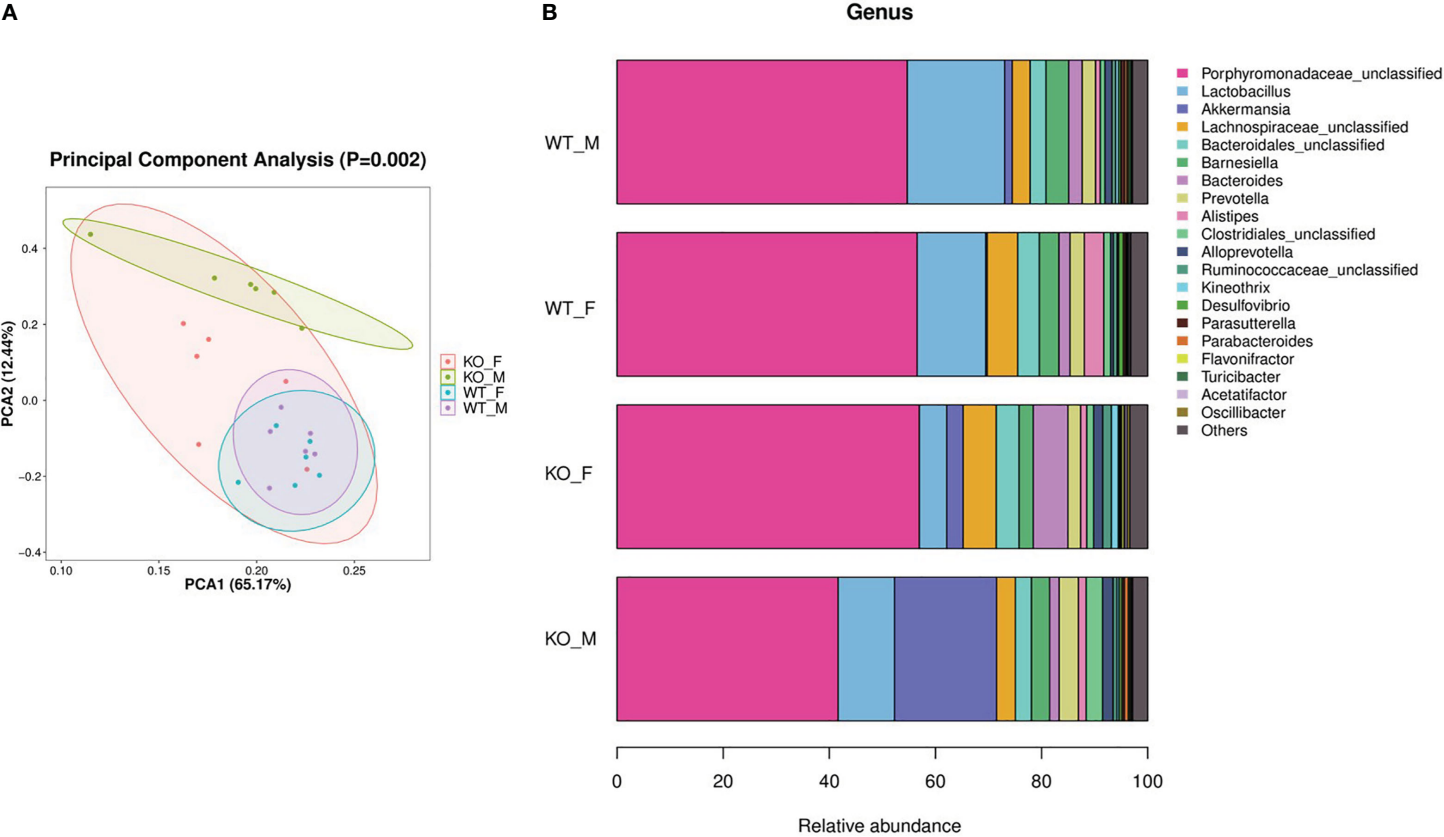
Pgam5 deficiency alters the gut microbiota in mice. Intestinal contents were collected from female and male adult *Pgam5^-/-^* and WT mice and total fecal microbiota profiles of colon were analyzed by bacterial taxa 16S rRNA amplicon sequencing. **(A)** PCA ordination plots with 95% confidence ellipses. **(B)** Microbiota composition of female and male adult *Pgam5* ^-/-^ and WT mice at the genus level for the 16S rRNA gene. The black dots mean outliers. KO-F, *Pgam5^-/-^* female; KO-M, *Pgam5^-/-^* male; WT-F, WT female; WT-M, WT male.

We collected intestinal contents from female and male adult *Pgam5^-/-^* and WT mice and analyzed the total fecal microbiota profiles. Principal component analysis (PCA) presented a trend of separation according to genotype (*p* = 0.002, **Figure 3A**). In both female and male mice, *Pgam5* KO was found to affect the diversity of gut microbiota, including a significant upregulation of *A. muciniphila* abundance (**Figures 3A, B**). The top three components of the gut microbiota were Porphyromonadaceae-unclassified, *Lactobacillus*, and *A. muciniphila* in mice, and the proportions of different microbiota varied among different sexes. For example, *A. muciniphila* was lower in WT female mice than in WT male mice, and *Pgam5* KO male mice had fewer Porphyromonadaceae-unclassified but higher *Lactobacillus* and *A. muciniphila* than KO female mice (**Figure 3B**). Interestingly, male *Pgam5* KO mice consistently exhibited high abundance of *A. muciniphila* and low Porphyromonadaceae-unclassified abundance (**Figure 3B**).

### FMT causes the alteration of the microbiota and metabolites

**Figure 4:**
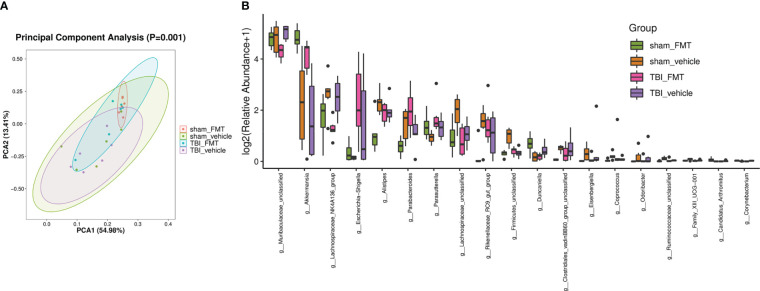
FMT causes the alteration of gut microbiota abundance. After FMT treatment in TBI mice, 16SrRNA sequencing was performed to analyze the gut microbiota in colon at 7 dpt. **(A)** PCA ordination plots with 95% confidence ellipses. **(B)** Barplot differential analysis of gut microbiota abundance is significantly different after FMT treatment. The Kruskal-Wallis test was used to test for species differences at the genus level, and significantly different genus were defined as *p* < 0.05.

FMT treatment may cause the reconstruction of intestinal flora in mice. We collected the intestinal tract of mice at 7 dpt of study 3, analyzed gut microbiota, and detected metabolites in the serum. PCA revealed a trend of separation by treatment (*p* = 0.001; **Figure 4A**). TBI induced the change of gut microbiota components, including a decrease of *Anaerotruncus*, Firmicutes-unclassified, *Lachnospiraceae-unclassified*, and *Ruminococcus-1* and increase of *Muribaculum* and *Gastranaerophilales-unclassified* (**Figure 4B**). Compared with the sham-vehicle group, sham-FMT mice presented more *A. muciniphila* and *Oscillibacter* and less Firmicutes-unclassified, *Lachnospiraceae-unclassified*, and *Ruminococcus-1* (**Figure 4B**). FMT resisted TBI-induced *Muribaculum* and *Gastranaerophilales-unclassified*, whereas the TBI-FMT group contained significantly higher *A. muciniphila* and low *Muribaculaceae-unclassified* relative to TBI vehicle (**Figure 4B**). The barplot differential analysis of gut microbiota abundance showed that *A. muciniphila* was significantly increased after FMT treatment in the sham group (**Figure 4B**). In addition, we identified taxa whose abundance differed significantly among the groups. These results indicated that differences could be identified at all taxonomic levels from the phylum to the genus and spread throughout all the phyla (**Figure 4**).

**Figure 5:**
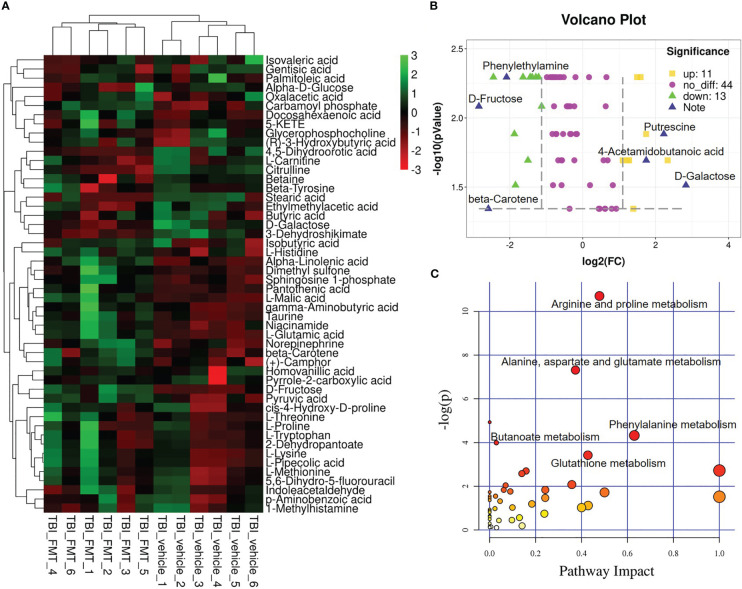
FMT alters serum metabolites in TBI mice. At 7 dpt, metabolites were detected in the serum. **(A)** Significant change of metabolite was shown by the heatmap in serum of TBI-vehicle and TBI-FMT mice, and the abundance of each metabolite was normalized by Z score normalization. **(B)** Volcano plot comparing the serum metabolites in TBI-vehicle and TBI-FMT mice. The yellow dot meant the upregulated metabolite and the green plot meant the downregulated metabolite (FC>2, *p <*0.05). **(C)** Pathway analysis was conducted on the different metabolites between TBI-vehicle and TBI-FMT mice.

FMT treatment caused the alteration of sugar, fatty acid, polyamines, and phenolic and phenyl derivatives, including a significant increase in D-fructose, β-carotene, norepinephrine, glutamic acid, carbamoyl phosphate, α-linolenic acid, retinoyl b-glucuronide, phenylethylamine, methyl jasmonate, and ophthalmate levels, and a decrease in D-galactose, methylmalonic acid, putrescine, 4-acetamidobutanoic acid, isocitric acid, 2-aminoacrylic acid, L-alanine, D-mannose, and 4-hydroxybenzoic acid levels. Top 50 abundant metabolites in TBI-FMT mice and TBI-vehicle mice were shown in **Figure 5A**. The volcano plot showed the up (n = 11) and down (n=13) metabolites, including phenylethylamine, D-fructose, D-galactose, 4-acetamidobutanoic acid, beta-carotene, and putrescine (**Figure 5B**). Possible metabolisms included alanine, aspartate, and glutamate metabolism (4-acetamidobutanoic acid, glutamic acid, alanine, carbamoyl phosphate), phenylalanine metabolism (phenylethylamine, phenylacetylglycine), butanoate metabolism (3-butynoate, γ-aminobutyric acid), glutathione metabolism (glutamic acid, glutathione, putrescine, ornithine), and arginine and proline metabolism (creatine, putrescine, 4-acetamidobutanoic acid) (**Figure 5C**). The data indicated that the FMT treatment maybe better maintenance of glutathione metabolism (an increase of glutamic acid and methionine) and a more hospitable environment in the periphery than in the TBI-vehicle group.

### FMT may embrace *Akkermansia muciniphila* to inhibit neuroinflammation and improves nerve injury after TBI

**Figure 6:**
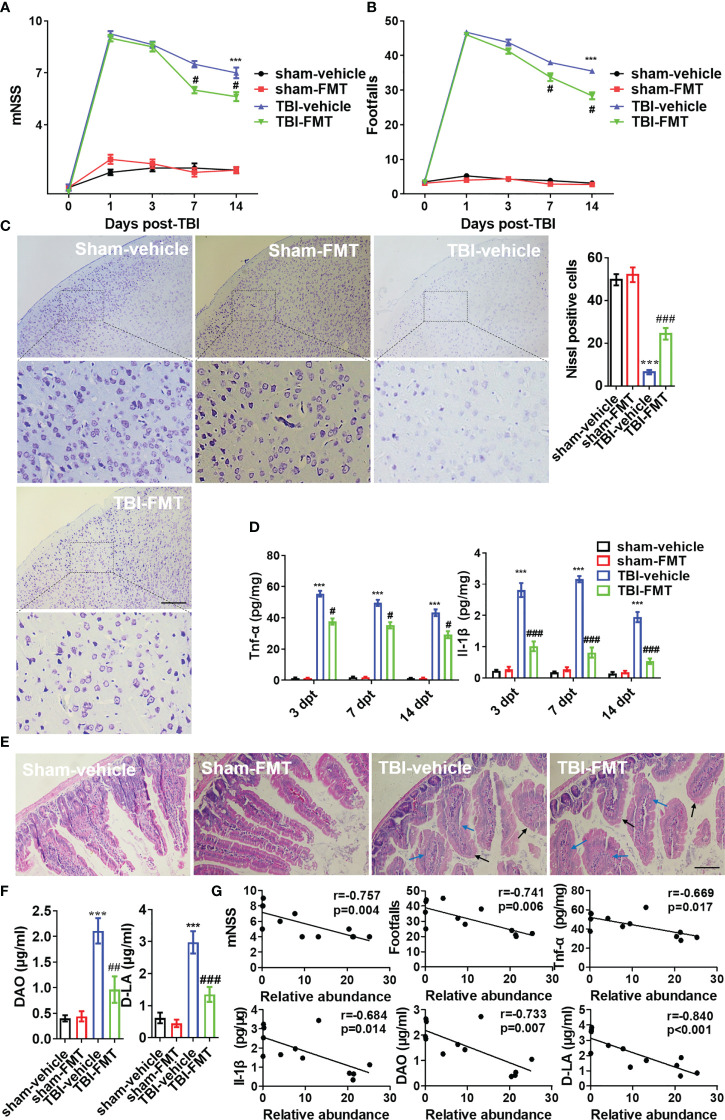
FMT alleviates neurological dysfunction and neuroinflammation post-TBI. After FMT treatment in TBI mice, behavioral tests, inflammatory cytokines, and histopathology were examined. Behavioral test was analyzed by mNSS **(A)** and beam walk test **(B)** at 1, 3, 7, and 14 dpt, n = 8. **(C)** Nissl staining was conducted to assess morphological condition and primary somatosensory cortex neuronal damage at 7 dpt. **(D)** ELISA analysis of Tnf-α and Il-1β in cortical tissue at 3, 7, and 14 dpt. **(E)** H&E staining analysis the damage of intestine at 7 dpt. Markers in the image indicated swollen (blue arrow) and smooth (black arrow) pathological tissues. **(F)** The levels of DAO and D-LA in serum were detected at 7 dpt. **(G)** The Pearson correlation analysis of *A*. *muciniphila* and mNSS, footfalls, Tnf-α, Il-1β, DAO, and D-LA in the TBI groups (TBI-vehicle and TBI-FMT). Data were shown as means ± SEM, n = 6, *** *p* < 0.001 *vs.* sham-vehicle group; # *p* < 0.05, ## *p* < 0.01, and ### *p* < 0.001, *vs.* TBI-vehicle group.

To confirm benefits of gut microbiota from *Pgam5* KO mice on nerve damage around the injury site, antibiotic-treated WT male mice in the vehicle and FMT groups were administered doses of 0.2 mL sterile PBS or FMT after TBI. All animals underwent motor function testing at 1, 3, 7, and 14 dpt. FMT did not cause behavioral abnormalities in the sham group (**Figures 6A, B**), but FMT minimized the increase in mNSS in TBI mice at 7 dpt (*p* < 0.05, **Figure 6A**), and the foot fault was significantly lower at 7 and 14 dpt in TBI-FMT group *vs.* those of TBI-vehicle group (*p* < 0.05, **Figure 6B**). The mNSS at 3 and 14 dpt were lower in TBI-FMT mice than that in TBI-vehicle group, but there was no statistical difference (**Figure 6B**). Brain histopathology is mainly concerned with primary somatosensory cortex around the injury site. Meanwhile, neuronal cell loss in primary somatosensory cortex was visibly better around the injury site (parietal cortex) in TBI-FMT group *vs.* that in TBI-vehicle group at 14 dpt (*p* < 0.05, **Figure 6C**). For Tnf-α and Il-1β analysis, FMT significantly reduced Tnf-α level (*p* < 0.05) and Il-1β level (*p* < 0.001) in cortical tissue at 3, 7, and 14 dpt (**Figure 6D**). Thus, treatment with FMT in male *Pgam5^-/-^* mice could suppress inflammatory factors and neuronal damage and improve neurological deficits following TBI.

After TBI and FMT treatment at 7 dpt, the intestinal mucosal injury was examined through pathology. H&E staining showed that the swollen (blue arrow) and smooth (black arrow) histopathology were increased after TBI (**Figure 6E**). The levels of DAO and D-LA were relatively high in TBI mice (*p* < 0.05, **Figure 6F**). It revealed intestinal mucosal structure and barrier function of mice were damaged at 7 dpt. However, FMT treatment resulted in negligible improvements in the structural change of intestinal microvilli, but less DAO level and D-LA level in serum of TBI-FMT mice than that in TBI-vehicle mice (**Figures 6E, F**). Furthermore, the Pearson correlation analysis of *A. muciniphila* and other indicators in the TBI groups (TBI-vehicle and TBI-FMT) was performed, which identified that *A. muciniphila* abundance was negatively correlated with the mNSS, foot fault, DAO level and D-LA level in serum, and Tnf-α level and Il-1β level in the cortical tissue (**Figure 6G**). In other words, *A. muciniphila* was negatively associated with intestinal mucosal injury and neuroinflammation after TBI, which may be beneficial for nerve injury.

### *Akkermansia muciniphila* improves neuroinflammation and nerve injury post-TBI via Nlrp3 inflammasome activation

**Figure 7:**
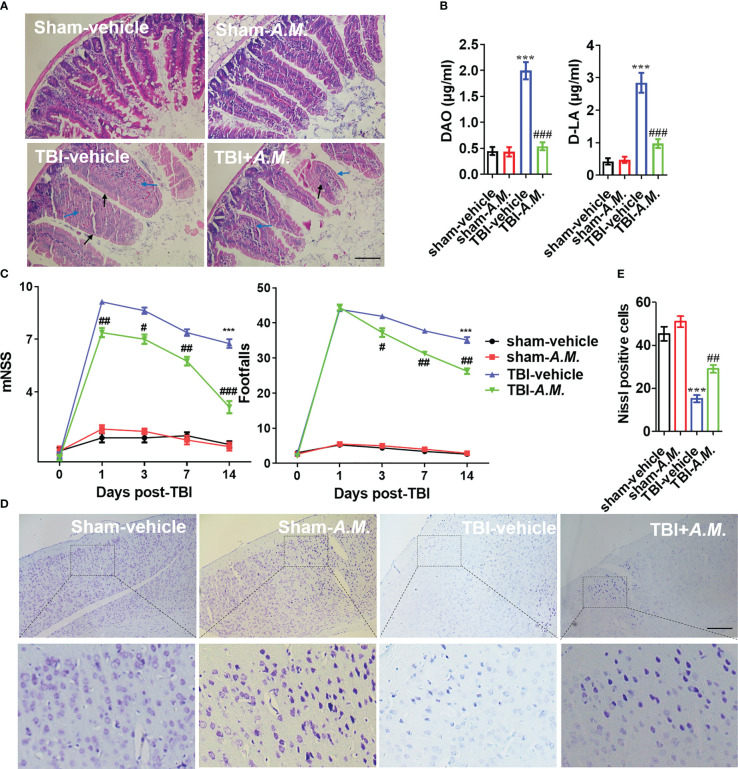
*A*. *muciniphila* exhibits an improvement of intestinal tract and neurological function in TBI mice. **(A)** H&E staining analysis the damage of intestine at 7 dpt. Markers in the image indicated swollen (blue arrow) and smooth (black arrow) pathological tissues. **(B)** The levels of DAO and D-LA in serum were detected at 7 dpt. **(C)** After FMT treatment in TBI mice, behavioral tests was analyzed by mNSS and beam walk test at 1, 3, 7, and 14 dpt, n = 8. **(D)** Nissl staining was conducted to assess morphological condition and primary somatosensory cortex neuronal damage at 7 dpt. **(E)** Quantitative analysis of the Nissl positive cells in primary somatosensory cortex at 7 dpt. Data were shown as means ± SEM, n = 6, *** *p* < 0.001 *vs.* sham-vehicle group; # *p* < 0.05, ## *p* < 0.01, and ### *p* < 0.001, *vs.* TBI-vehicle group.

**Figure 8:**
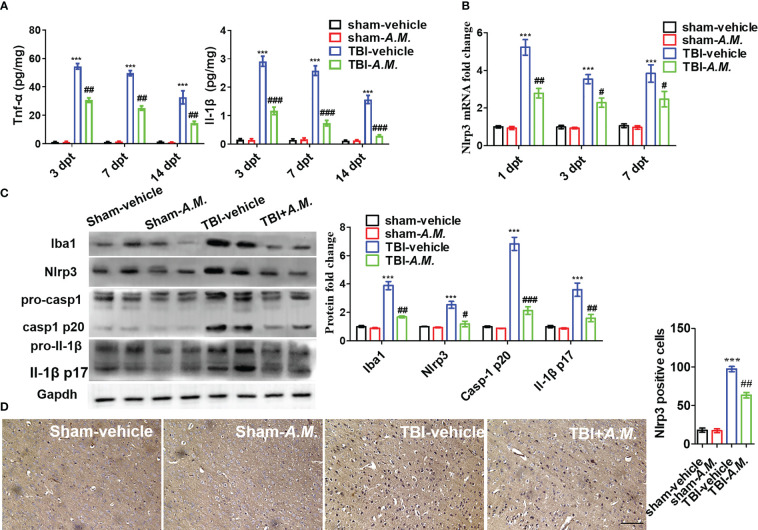
*A*. *muciniphila* decreases neuroinflammation via Nlrp3 inflammasome activation following TBI. After *A*. *muciniphila* transplantation, inflammatory cytokines and Nlrp3 inflammasome activation were measured in cerebral cortical tissue. **(A)** ELISA analysis of Tnf-α and Il-1β in cortical tissue at 3, 7, and 14 dpt. **(B)** qPCR analysis of *Nlrp3* mRNA level at 1, 3, and 7 dpt. **(C)** WB analysis of Iba1, Nlrp3, caspase-1, and Il-1β expression in the contusion location at 7 dpt. **(D)** IHC analysis of Nlrp3 at 7 dpt. Scale bar: 20 μm. Data were shown as means ± SEM, n = 6, *** *p* < 0.001 compared with the sham-vehicle group; # *p* < 0.05, ## *p* < 0.01, and ### *p* < 0.001, compared with the TBI-vehicle group. *A.M.*, ***(A)*** *muciniphila.*.

To examine the neuroprotection of *A. muciniphila* in, we administered gavage doses of 0.2 mL sterile PBS or *A. muciniphila* to WT male mice with TBI. At 7 dpt, *A. muciniphila* treatment reduced intestinal mucosal injury (including the swollen and smooth histopathology) (**Figure 7A**) and DAO level and D-LA level in serum (**Figure 7B**). After *A. muciniphila* treatment, the neurofunctional score and foot fault were significantly better in the *A. muciniphila* group compared with that in vehicle group at 3, 7, and 14 dpt (*p* < 0.05, **Figure 7C**). Nissl staining showed that primary somatosensory cortex neurons loss around the injury site (parietal cortex) was also suppressed by *A. muciniphila* treatment at 14 dpt (**Figures 7D, E**). In Tnf-α and Il-1β detection, *A. muciniphila* significantly reduced Tnf-α level (*p* < 0.01) and Il-1β level (*p* < 0.001) in cortical tissue at 3, 7, and 14 dpt (**Figure 8A**). TBI promoted Nlrp3 inflammasome activation in cortical tissue, and *A. muciniphila* treatment suppressed *Nlrp3* mRNA level (**Figure 8B**) and Iba1, Nlrp3, caspase-1 p20, and Il-1β p17 expressions in cortical tissue (**Figure 8C**). Furthermore, immunohistochemistry results demonstrated that TBI-induced Nlrp3 positive stain was decreased following treatment with *A. muciniphila* (**Figure 8D**). The findings suggest *A. muciniphila* in gut microbiota may significantly ameliorate neuroinflammation and nerve injury by regulating Nlrp3 inflammasome activation in mice with TBI.

## Discussion

**Figure 9:**
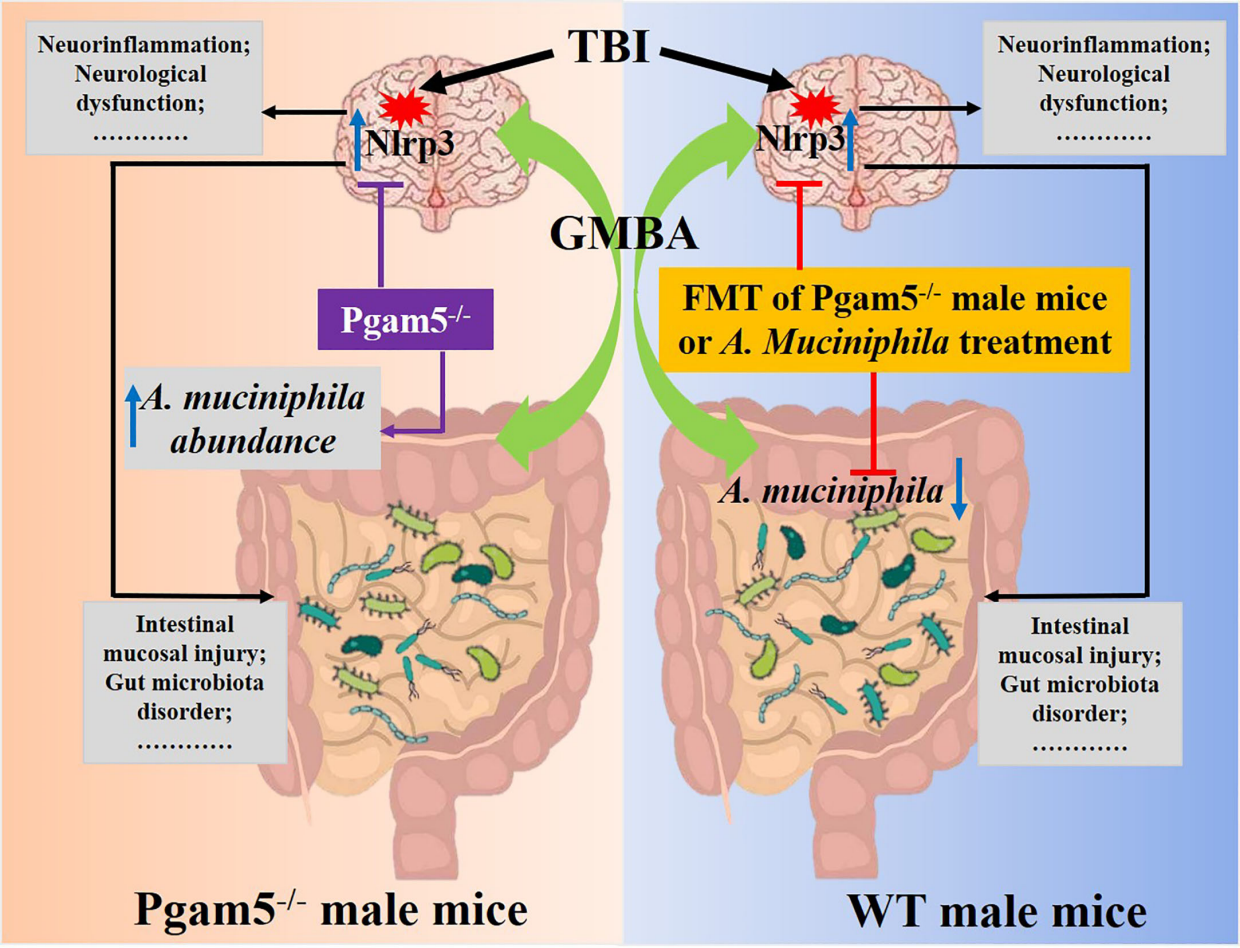
Schematic diagram showing that *A. muciniphila*-Nlrp3 is involved in the neuroprotection of *Pgam5* deficiency in TBI mice. Male *Pgam5* knockout mice consistently exhibited an increased abundance of *A. muciniphila* and *Pgam5* deficiency improved neuroinflammation and neurological deficits. FMT of *Pgam5^−/−^* male mice suppressed neuroinflammation and improved neurological deficits, and *A. muciniphila* abundance was negatively associated with intestinal mucosal injury and neuroinflammation post-TBI. Moreover, *A. muciniphila* treatment ameliorated neuroinflammation and nerve injury by regulating Nlrp3 inflammasome activation in mice with TBI.

The academic community has been devoted to exploring various neuroprotective strategies after neuronal injury (29–31). In present work, we found significant differences in gut microbiota composition (including a high level of *A. muciniphila*) in male *Pgam5^-/-^* mice compared to male WT mice. The inhibition of the gut microbiota (by antibiotic treatment) reduced the neuroprotective function of *Pgam5* deficiency in the early stages of TBI, and the transplantation of the gut microbiota from male *Pgam5^-/-^* mice also improved neuroinflammation and neurological dysfunction post-TBI. Furthermore, treatment with *A. muciniphila* by gavage alleviated TBI-induced inflammatory factor levels (Tnf-α and Il-1β), neuronal-loss, and neurological dysfunction by suppressing Nlrp3 inflammasome activation. The data highlighted *A. muciniphila* as a potentially beneficial factor in the neuroprotection of *Pgam5* deficiency after TBI based on its effects on neuroinflammation via suppressing Nlrp3 inflammasome activation in mice (**Figure 9**).

Studies have reported that alteration of genotypes also regulates the gut microbiota (32, 33). Zhang et al. reported that *Nlrp3* KO reduces abundance of *Bacteroides* but enhances abundance of *Desulfovibrio*, *[Ruminococcus]*, *Mucispirillum*, *Oscillospira*, *[Prevotella]*, and *Ruminococcus* (32). *Fto* KO mice consistently exhibit a high abundance of Lactobacillus, a low abundance of unclassified Porphyromonadaceae, and *Helicobacter*, which contributed to decrease depression- and anxiety-like behavior in mice (33). We found that *Pgam5* KO male mice contained fewer Porphyromonadaceae-unclassified and higher *Lactobacillus* and *A. muciniphila* than KO female mice. Mitochondria in intestinal epithelial cells participates in intestinal cell proliferation and cell cycle regulation, which mediates intestinal barrier and gut microbiome (34, 35). And activation of Drp1 interferes with gut microbiome abundance and short-chain fatty acid production in a reactive oxygen species (ROS)-specific manner, thus severely disturbing tight junction and intestinal barrier following hemorrhagic shock (26). As PGAM5 mediates Drp1 activation, mitochondrial function, and metabolism, *Pgam5* KO may disturb microbiological compositions in *A. muciniphila* phylum via mitochondria-mediated intestinal barrier and gut microbiome. FMT from *Pgam5^-/-^* male mice increased *A. muciniphila* level in WT male mice, which also alleviated gut microbiota disorders (including a decrease of *Anaerotruncus*, Firmicutes-unclassified, *Lachnospiraceae-unclassified*, and *Ruminococcus-1* and an increase of *Muribaculum* and *Gastranaerophilales-unclassified*), thereby improving TBI-induced intestinal mucosal injury and nerve injury by suppressing TLR4/NF-κB signaling. The data fit well with previous evidence that the levels of traditionally beneficial bacteria (Firmicutes, *Lachnospiraceae*, *Ruminococcus*) and pathogenic bacteria (*Proteobacteria*) are correlated with lesion size due to TBI (6, 11, 12, 14, 19). Treangen et al. reported that FMT from mice with TBI leaded to a noteworthy reduce in *Lactobacillus gasseri*, *Ruminococcus flavefaciens*, and *Eubacterium ventriosum* (13). Nicholson et al. pointed out that a reduced ratio of Firmicutes to Bacteroidetes may be a result of the stress response after TBI (14). Limited clinical evidence from a recent investigation suggests that multiple trauma patients show a reduce in good bacteria, but a rise in *Clostridiales* and *Enterococcus* within 72 h following severe trauma (12). Management of the gut microbiota is intricate and poorly understood, whereas many clinical samples with elaborate injury degrees and stages may drive the diagnosis and treatment of the gut microbiota as biomarkers. However, delineating the exact mechanism of *Pgam5* deficiency regulating the gut microbiota warrants further studies.

Metabolites also serves as potential diagnostic and therapeutic biomarkers for multiple trauma (6). Chitturi et al. reveal a series of differential metabolites at 72 h post-TBI in Sprague-Dawley rats (36, 37), and TCA cycle metabolism maintains brain energy metabolism (37). Holshouser et al. perform three-dimensional proton magnetic resonance spectroscopic imaging in pediatric subjects with injury and demonstrate that a decrease in N-acetylaspartate level is an early indicator and measurement in subcortical area is more predictive of long-term cognitive outcomes post-TBI (38). Some unique and intricate alterations in hippocampal metabolites have been identified in two TBI-related pathways, taurine and hypotaurine metabolism, and arginine biosynthesis (5, 39). We further assessed the metabolites in the serum and verified that TBI resulted in tryptophan metabolism (less abundant of serotonin) and aberrant TCA cycle in the periphery, and FMT treatment from *Pgam5^-/-^* mice was better able to maintain glutathione metabolism (including enhancing glutathione metabolism (increase of glutamic acid and methionine) 7 days following TBI. FMT treatment upregulated glutamic acid and methionine levels in plasma of TBI mice, which were high abundant metabolites (top 50) in plasma and might be associated with glutathione synthesis. Mechanically, unregulated release of glutamate cannot be effectively buffered or cleared, resulting in impaired glutamate level in extracellular space, which may lead to activation of cell death pathways and secondary injury after acute TBI (40). However, recently report demonstrates glycine and glutamic acid alleviate H_2_O_2_-induced oxidative stress via increasing superoxide dismutase and glutathione peroxidase activities of bacteria, which may provide new insights of glutamic acid in cellular antioxidant activity (41). Methionine is involved in the synthesis of cysteine, which is a key rate-limiting for glutathione (42). Arun et al. demonstrate blast exposure decreases methionine level and disrupts in methionine metabolism elicited by might prominently contribute to neuronal injury by suppressing cysteine and glutathione synthesis to promote sustained oxidative stress, in blast-induced TBI rat (43). Thus, glutathione level and antioxidant response were important part of the role of FMT (from *Pgam5^-/-^* male mice) after TBI.

Because of the variation in *A. muciniphila* abundance after FMT treatment, further analysis showed that *A. muciniphila* abundance was negatively correlated with intestinal mucosal injury and neuroinflammation post-TBI. *A. muciniphila* is extensively colonized in the human intestinal mucosa (15, 16) and has been considered a promising candidate for probiotics (17, 18). Decreased *A. muciniphila* abundance is a risk factor for diabetes, obesity, appendicitis, inflammatory bowel disease, and other metabolic syndromes (44–49), and its abundance has been related to the metabolic status of human health, particularly in terms of fasting blood glucose, plasma triglycerides, and body fat distribution (48). Hou et al. show *Akkermansia* is significantly enriched in the patients with TBI and it increase at 3 days in surgical brain injury (SBI) rat, but the abundance of *Akkermansia* is decrease at 7 days and the mean is basically flat with healthy control; Furthermore, the oral administration of probiotics increases *Akkermansia* abundance at 3 days in SBI rat (in the supplementary results) (50). Li et al. find *Akkermansia* significantly increases in ischemic stroke patients (51), but some reports show *Akkermansia* decreases in stroke patients and radiation-induced brain injury mice (52–54). The cognitive disorder in Alzheimer’s disease (AD) is related to *Akkermansia* abundance, and supplementing the diet with the *Akkermansia* protects against the cognition impairment (55). Our data showed *Akkermansia* abundance was noteworthy down-regulated after TBI mice, which was different from some previous reports and may be due to difference of design and method, as well as the differences of individual animals, fecal sample collection time and feeding environment in this study. Such inconsistent changes in gut microbiota and metabolites have been reported in some previous studies. Therefore, more experiments with large samples are expected to be further explored.

Yaghoubfar et al. have reported *A. muciniphila* and its extracellular vesicles (EVs) administration enhance serotonin level and Il-10 mRNA level but EVs decreases Tnf-α mRNA level in the colon; however, it seems that they may not cause colonic inflammation in male C57BL/6J mice (56). Moreover, oral gavage with 10^9^ CFU/200 μL *A. muciniphila* reverses high fat obesity through improving intestinal barrier integrity, inflammation, energy balance, and triglyceride and glucose in the blood (57), which also suppresses mRNA expression of Tlr4 and Il-6 but not Tnf-α in adipose tissues (57). Ashrafian et al. also find *A. muciniphila* and its EVs may be paraprobiotic and postbiotic agents and prevents obesity via regulating gut-adipose-liver axis (58). We demonstrated that oral gavage with *A. muciniphila* suppressed microglia and Nlrp3 inflammasome activation in the brain, which improved neuroinflammation and nerve injury post-TBI. Although evidence has emerged that neurodegeneration is strongly associated with the gut microbiome composition, the mechanism of *A. muciniphila* on neurological problems and CNS diseases is limited. Preliminary and clinical evidence associates *A. muciniphila* to multiple sclerosis (MS) (59, 60), PD (61, 62), AD (55, 63), stroke (64), epilepsy (65), and amyotrophic lateral sclerosis (ALS) (66) and increased *A. muciniphila* abundance in MS, PD, and stroke patients and APP/PS1 mice (60, 61, 63, 64). Long-term administration of *A. muciniphila* effectively improves blood glucose and serum diamine oxidase levels, intestinal barrier dysfunction, Aβ 40-42 disposal in cerebral cortex, and spatial learning and memory in mice with AD (55). Ketogenic diet (KD)-associated *A. muciniphila* and *Parabacteroides* mediate and confer antiepileptic function of the KD by modulating amino acid γ-glutamylation and hippocampal gamma aminobutyric acid/glutamate (65), but purified *A. muciniphila* has no antiepileptic action via crosstalk with other bacteria (65). This indicates that the biological function of *A. muciniphila* depends not only on the direct action of outer membrane surface molecules but also on the interaction with other gut microbiota. *A. muciniphila* ameliorated the symptoms of ALS associated with nicotinamide accumulation in the CNS of *Sod1-Tg* mice (66). Low *A. muciniphila* abundance is also negatively associated with high circulatory HMGB1 level, blood-brain barrier (BBB) disruption, and neuroinflammation in Gulf War illness, which may be attributed to NLRP3 inflammasome activation (67). The regulatory network of NLRP3 in nerve injury has attracted much attention (68–70), and DAMPs, potassium and chloride efflux, sodium and calcium efflux, altered calcium signaling, lysosomal instability, and products of mitochondrial dysfunction such as mitochondrial DNA and ROS, are known to initiate NLRP3 inflammasome post-TBI (67). *A. muciniphila* improves neuronal function via inhibiting Nlrp3 inflammasome activation, because Nlrp3 inflammasome is a major driver of neuroinflammation and neurobehavioral disturbances following TBI and that is regarded as a potential a biomarker and therapeutic target (71). Reports have demonstrated that intestinal dysbiosis augments disease progression via NLRP3 (72, 73), and bacterial products and bile acids can induce NLRP3 inflammasome activation (72, 74). So, *A. muciniphila* improves neuronal function via inhibiting Nlrp3 inflammasome-mediated neuroinflammation, which may also involve in the regulation of priming and activation of NLRP3 inflammasome by metabolites. However, we did not identify bile acids, nicotinamide, γ-glutamylation, and glutamate in our samples, and we were unable to dissect whether decreased Nlrp3 activation is mediated by a direct increase of *A. muciniphila* or a consequence of changes in the metabolite of *A. muciniphila*. Thus, the mechanism of interaction between metabolites and Nlrp3 inflammasome activation will be investigated in further works, and whether *A. muciniphila*, its extracellular vesicles, its membrane protein, or its secretory protein plays a key role is very interesting research in Nlrp3 inflammasome activation that deserves further discussion.

## Conclusion

In conclusion, the work was the first to investigate intestinal microbial population in *Pgam5^-/-^* mice, providing evidence for involvement of PGAM5 in the regulation of gut microbiota in neuroinflammation and nerve injury by embracing *A. muciniphila*-Nlrp3 to contribute to peripheral effects. We propose *that A. muciniphila* benefits TBI outcomes, which requires further exploration of the negative effects in specific scenarios. Moreover, the performance of global gene KO animal models involves the influence of peripheral, particularly, gut microbiota on target tissue or cell functions, but cannot be completely excluded or ignored. Considering our findings, this study provided new insights and revealed pathological changes of acute TBI in *Pgam5* deficient mice and the potential benefits of *A. muciniphila* in mice with TBI, which may help to more accurately identify the potential treatment outcome of TBI.

## Data availability statement

The datasets presented in this study can be found in online repositories. The names of the repository/repositories and accession number(s) can be found below: PRJNA741415 (SRA).

## Ethics statement

All *in vivo* experiments were approved by the Animal Care and Use Committee of Xi’an Peihua University, China, and performed according to the recommendations of the Guide for the Care and Use of Laboratory Animals.

## Author contributions

YC, QX, and KX planned and designed the research. HW, KG, and TL performed CCI model and histological evaluation. JC, HW, and KG performed antibiotic treatment, FMT, and experiments of molecular biology. LY contributed to the statistical analysis of behavioral analysis. JM and KX provide the statistical analysis and analysis of correlation between the relative indexes and *Akkermansia muciniphila* level. JC, HW and JH were responsible for contribution to the animal behavioral tests. YC wrote the original draft and ZW contributed to review and editing. YC, JC, and KX refined the review comments. JQ and KX edited the proof. All authors contributed to the article and approved the submitted version.
